# The Role of Campus as an Urban Multiuse Protected Area in Bird Nocturnal Roosting Habitat Function

**DOI:** 10.1002/ece3.72554

**Published:** 2026-01-25

**Authors:** Meng He, Bo Li, Wei Du, Chunlan Du

**Affiliations:** ^1^ Faculty of Architecture and Urban Planning Chongqing University Chongqing China; ^2^ Key Laboratory of New Technology for Construction of Cities in Mountain Area Chongqing University Chongqing China

**Keywords:** biodiversity conservation, bird nocturnal roosting habitat, campus green space, green space function, landscape ecology

## Abstract

Campus green spaces are vital components of urban green infrastructure, providing crucial habitats for wildlife, especially birds, in fragmented urban environments. However, their role in supporting nocturnal bird habitats remains underexplored. This study investigates nocturnal bird roosting habitat selection in the green spaces of a university campus in western China. The seasonal surveys were conducted along fixed routes to track the distribution of birds' nocturnal roosts on campus. Infrared hotspots were detected using thermal imaging night‐vision cameras. Roosting tree species were identified, and roosting point heights were estimated visually. The survey results identified eight nocturnal roosting sites and common campus vegetation, which were used for in‐depth analysis of bird roosting habitat characteristics. The study found that: (1) Campus birds preferred *Ficus concinna* and *Ficus virens* (93% selection rate), especially those with dense foliage and high concealment. (2) Birds showed seasonal variations in their roosting positions. In summer, they were more dispersed, while in autumn and winter, they concentrated vertically. Horizontally, they preferred the outer ends of branches away from the trunk. (3) The number of infrared (thermal) hotspots corresponding to birds' nighttime roosts was significantly higher in summer than in the other seasons. Based on these findings, the paper proposes strategies to optimize campus green space management, including the targeted planting of tree species, adjustments to vegetation structure, and the reduction of excessive branching to enhance nocturnal roosting sites. This research offers valuable insights into the nocturnal behavior of urban birds and provides practical recommendations for urban planners and conservationists to promote biodiversity‐friendly urban design and management policies.

## Graphical abstract

Campus green spaces are essential for providing critical nocturnal habitats, playing a key role in supporting urban bird species in fragmented environments. Seasonal variations significantly influence nocturnal bird roosting site selection, with birds adapting their roosting patterns to changing environmental conditions throughout the year. Nocturnal birds preferentially select Ficus species with dense foliage and high concealment, which provide enhanced shelter and protection from predators. In summer, birds exhibit more dispersed roosting patterns to optimize microclimate conditions, while in autumn and winter, they concentrate in more sheltered, secure areas. To support nocturnal roosting, campuses should retain tree branches of varying sizes and heights, maintain diverse vegetation, and minimize disturbances.

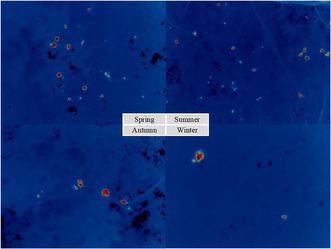

## 1 Introduction

Urban green spaces provide essential habitats for wildlife, playing a crucial role in biodiversity conservation (Davis and Glick 1978). However, accelerated urbanization has led to habitat fragmentation and declining habitat quality, posing significant threats to biodiversity (Zhu et al. 2020). Birds, as sensitive indicators of environmental change (Sulaiman et al. 2013), play a key role in assessing urban habitat quality and biodiversity (Callaghan et al. 2018). Consequently, they are frequently studied, including in research on ecological habits (Wang and Chu 2021), distribution characteristics (Sandström et al. 2006), temporal dynamics (Chen et al. 2023), and environmental factors influencing urban bird habitats (Da Silva et al. 2021).

While university campuses primarily serve educational purposes, they are also crucial components of urban green space and hold significant value for bird diversity conservation (Zhang et al. 2018). Cerwinka's (2019) survey at the University of Pennsylvania showed that campuses offer vital stopover habitats for migratory birds and provide resident birds with foraging and nesting sites. Campus green spaces offer birds foraging, refuge, and roosting sites (Rajashekara and Venkatesha 2017; Xiao et al. 2024). Additionally, these spaces provide students, faculty, and the public with direct interactions with nature, while pleasant soundscapes like birdsong can boost positive emotions (Chen et al. 2022).

Current campus management often overlooks the effective protection of biological habitats, especially for birds (Elmqvist et al. 2013). For example, improper plant management and frequent pruning can negatively affect bird diversity (Dearborn and Kark 2010). Such practices may weaken campus green spaces' ecological role in supporting bird diversity. Scientific planning, design, and management are essential to support campus green spaces' ecosystem services in education and bird conservation, but research on this remains limited.

These habitats are essential for bird populations and, to some extent, influence the overall status of bird diversity in the region (Gou et al. 2022). While the term “nocturnal habitat” is sometimes used in the literature, we focus here on the roosting sites used by diurnal birds during the night. In this study, we did not target nocturnal bird species; instead, we surveyed diurnal birds at their nighttime roosts. To avoid confusion, we use the term “birds' nighttime roosts” throughout. A number of scholars have studied the nocturnal habitats of birds and their nocturnal behaviors (Prior and Weatherhead 1991; Smith et al. 2008). For example, Liao et al. (2008) examined the nocturnal behaviors of the *Arborophila rufipectus* in Laojunshan Nature Reserve, and Wang and Chu (2021) studied how seasonal changes affect the nocturnal roosting behavior of urban cockatoos regarding microhabitat characteristics. Generally, most studies focus on nature reserves or, less often, urban green spaces and are typically species‐specific. However, research on the role of campus green spaces as nocturnal habitats supporting bird biodiversity is scarce.

Understanding nocturnal roosting site selection by birds in campus green spaces is crucial for optimizing green space management and enhancing the functional assessment of urban ecosystems. This study investigates birds' nighttime roosts within the green spaces of Chongqing University to quantify temporal and spatial variation in roost‐site selection, identify key factors influencing roosting site choice, and propose management strategies to enhance the campus's ecological value for biodiversity conservation.

## 2 Methods

### 2.1 Study Site

**FIGURE 1:**
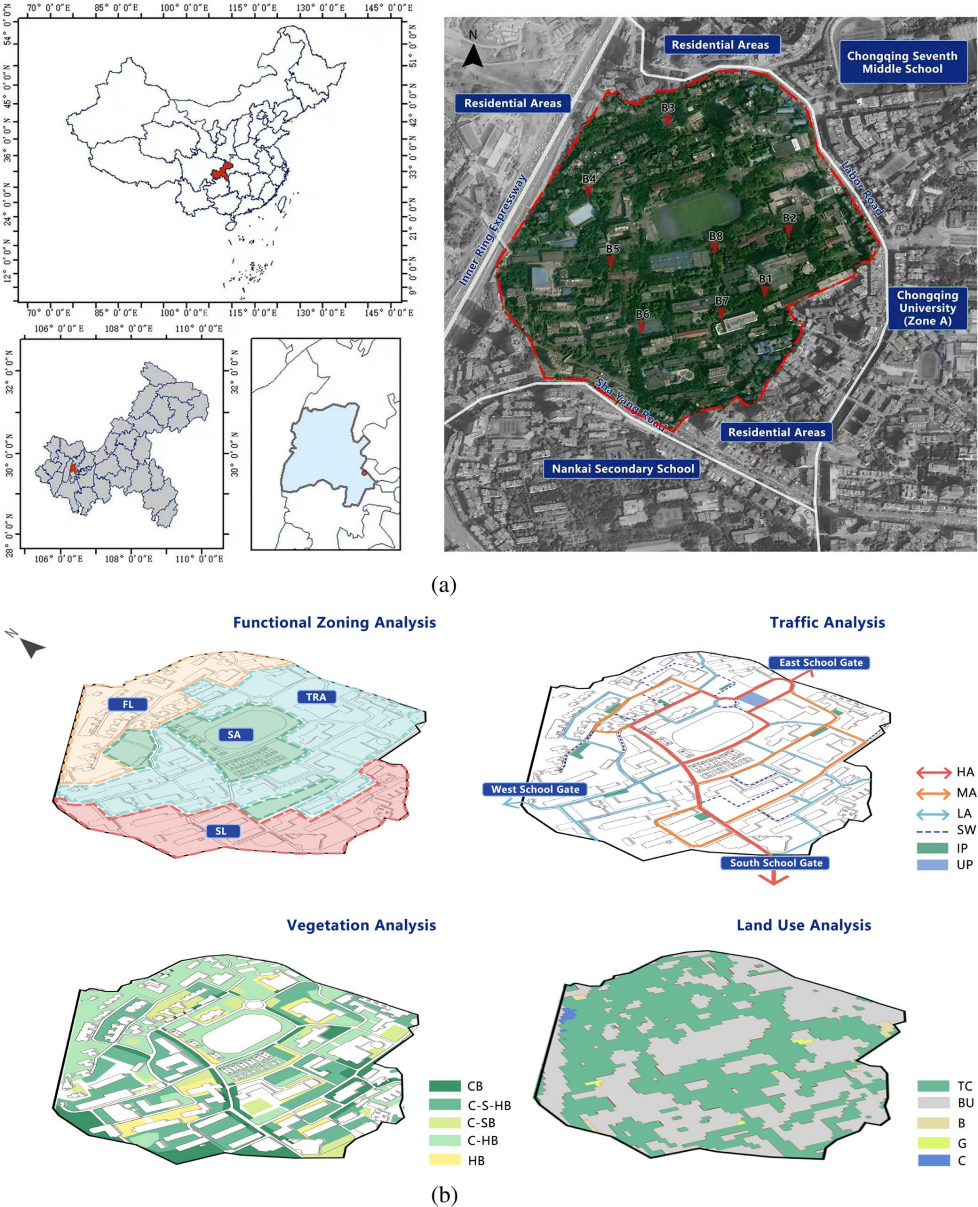
Campus use of space and vegetation analysis. (a) Geographic location of the study site in Chongqing, China, and satellite image of the campus with boundary (red line) and eight sampling plots (B1–B8), adjacent to residential areas and schools. (b) Functional zoning (SA, sports area; TRA, teaching and research area; SL, student living area; FL, faculty living area), traffic hierarchy (HA, high‐traffic artery; MA, medium‐traffic artery; LA, local access; SW, sidewalk; IP, internal path; UP, underground passage), vegetation types (AB, arbor–bush; A‐S‐HB, arbor–shrub–herbaceous; A‐SB, arbor–shrub; A‐HB, arbor–herbaceous; HB, herbaceous), and land‐use categories (TC, teaching/cultural; BU, buildings; B, bare land; G, green space; C, circulation).

The study area is located within Campus B of Chongqing University, a prominent university in Chongqing, situated in the Shapingba District (29°33′46″ N, 106°28′10″ E, Figure 1). The region experiences an annual average temperature of approximately 17.4°C, with January, the coldest month, averaging 7.8°C, and July, the hottest month, averaging 28.5°C. The area also has a rainy season primarily spanning from May to September. Annual precipitation ranges between 1, 000 mm and 1, 300 mm. Campus B encompasses an area of roughly 33.556 ha and is endowed with rich green spaces, predominantly characterized by tree species such as *Ficus virens*, *Cinnamomum camphora* , and *Ficus concinna*. The overall greenery coverage rate reaches 54.8%. According to functional distinctions, the campus is divided into four main areas: the Faculty and Staff Living Area, Student Living Area, Teaching and Research Area, and Sports and Recreation Area (Table 1). Eight survey points (B1–B8) were established across four functional zones, each with distinct habitat and disturbance profiles. Points B1 and B2, located in the core teaching area adjacent to main roads, experience high‐intensity human commuting and feature manicured, functional greening. The northern points B3 and B4 (Faculty Living Area) offer quieter conditions with more natural vegetation. Points B5 and B7, situated at the periphery of the teaching zone, represent a transition from functional to natural greening. The western point B6 (Student Living Area) is characterized by medium‐intensity, circulation‐dominated activity. The southern point B8 (Sports Area) is dominated by extensive hard pavement and high‐intensity sports activities, representing a highly disturbed, open habitat.

**TABLE 1:** Basic overview of different functional zones.

Functional partition	Area/m^2^	Frequency of evening activities (times/h·observation point)	Typical activities	Activity intensity	Vegetation structure and type	Includes sample plots
Faculty and staff living area	90,972	133	Strolling, commuting, walking dogs	Low	Natural greening; arbor + herbaceous vegetation	B3\B4
Student living area	72,393	74	Commuting, walking	Medium	Functional greening oriented toward circulation; mixed structure of trees and herbs	B6
Teaching and research Area	158,124	452	Commuting, walking	Medium	Functional greening; mixed tree‐herbaceous structure with guidance‐based layout	B1\B2\B5\B7
Sports and recreation area	40,030	122	Strolling, Ball games, jumping exercises	High	Dominated by hard pavement and partially covered by arbor vegetation	B8

### 2.2 Data Collection

Birds generally enter a resting state at night, during which their activity levels significantly decrease. The nocturnal roosting locations are typically established after sunset and remain stable until birds resume activity at sunrise the following day. Identification of roosting trees was primarily conducted shortly after sunset or just before dawn, when birds had settled into their nocturnal resting locations.

#### 2.2.1 Survey Timing and Frequency

Field surveys for this research were conducted from August 2018 to July 2019, encompassing both bird roosting behavior observations and vegetation surveys within selected plots. To systematically capture seasonal differences in bird roosting behavior, surveys were carried out in four representative months: spring (May), summer (August), autumn (November), and winter (January of the following year). Each month, surveys were conducted continuously for three consecutive days. Monitoring began approximately 1 h after sunset and lasted for 2–3 h, a period when birds generally enter and stabilize at their roosting sites. To control for potential inaccuracies arising from nocturnal movement, supplementary observations were occasionally conducted just before dawn.

#### 2.2.2 Survey Routes and Plot Selection

Multiple survey routes were planned along the primary road network on the campus to ensure coverage of different vegetation types and potential bird activity areas. Observers moved slowly along the predetermined routes while systematically scanning the vegetation on both sides. Necessary foot surveys were supplemented for internal green spaces not fully visible from the roads to minimize observational gaps. Areas with conspicuous nocturnal bird aggregations and representative vegetation structures were identified as fixed study plots through initial reconnaissance.

The measurement of vegetation parameters focused on vegetation type and vertical coverage. Within the selected plots, all major tree species were identified in situ during daytime. Identification was based on key morphological characteristics such as leaf morphology and bark texture, with reference to authoritative taxonomic works like Flora of China. The percent cover (%) of the tree layer, shrub layer, and herbaceous layer was visually estimated to quantify the three‐dimensional vegetation structure.

#### 2.2.3 Equipment and Observation Procedures

**FIGURE 2:**
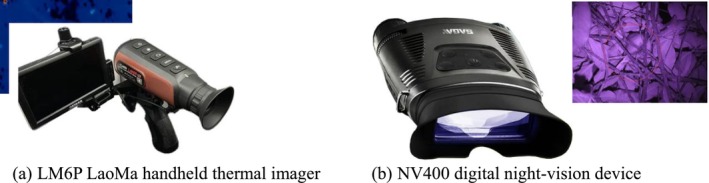
Instruments used in nocturnal surveys. (a) LM6P handheld thermal imager; inset shows canopy thermal “hotspots.” (b) NV400 digital night‐vision device; inset shows the verified outlines/positions of roosting birds under low light.

During data collection, an LM6P thermal imager was employed to scan individual trees and shrubs along each survey route. A night vision device was used to confirm the presence of birds at nighttime roosts (hotspots) (Figure 2). The detailed operational procedures were as follows:

##### 2.2.3.1 Multi‐Angle Detection

Observations were conducted from a suitable distance (approximately 5–10 m or adjusted according to field conditions), scanning individual tree crowns from multiple angles, from bottom to top, to minimize missed detections due to foliage obstruction. Potential roosting bird locations were identified by real‐time “hotspots” on the thermal imaging screen.

##### 2.2.3.2 Hotspot Verification

Upon detecting a hotspot, a night vision device was immediately used to confirm the hotspot's outline and activity. In cases of uncertainty, brief illumination with a flashlight was employed to further verify the presence of birds while minimizing disturbance. Subsequently, the tree species or specific branch location associated with the hotspot was recorded.

##### 2.2.3.3 Recording Standards

Confirmed bird roosting locations were documented, including time, specific location, tree species or vegetation type, estimated height, and the number of bird hotspots. Due to operational challenges posed by nighttime conditions and the specific nature of nocturnal roosting (e.g., high numbers of roosting birds, dense foliage), direct measurement of roosting height was difficult. Therefore, the heights of roosting branches were visually estimated (Hou et al. 2012). Although visual estimation may introduce potential errors, consistency was ensured by repeated calibrations and observations conducted by the same researcher, providing valid reference data for studying spatial patterns of bird nocturnal roosting.

### 2.3 Data Analysis

This study employed multiple statistical models to examine the effects of various factors on roosting behavior, complemented by visualization techniques to interpret and present the results. First, a one‐way analysis of variance (ANOVA) was used to test for seasonal differences in roosting height; as the assumption of homogeneity of variances was violated (Levene's test, *p* < 0.05), Tamhane's T2 post hoc test, which does not assume equal variances, was applied for pairwise comparisons between seasons. Second, a generalized linear model (GLM) was employed to analyze the count data of roosting hotspots. Due to detected overdispersion in an initial Poisson model, a negative binomial distribution was ultimately selected for the final model, which included the main effects of tree species and season, as well as their interaction term. Furthermore, to intuitively reveal spatial patterns and statistical trends, the spatial distribution of roosting sites was mapped using ArcGIS, and various statistical graphs, including boxplots, Sankey diagrams, and stacked bar charts, were generated using OriginPro.

## 3 Results

### 3.1 Nocturnal Observations and Spatial Distribution of Campus Birds

**FIGURE 3:**
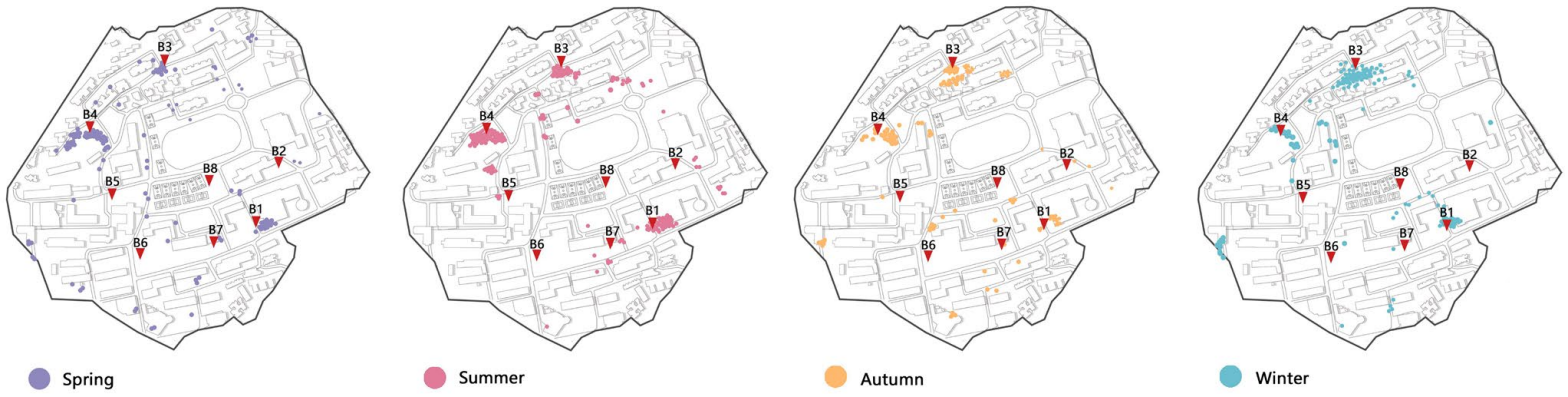
Seasonal spatial distribution of thermal hotspots corresponding to birds' nighttime roosts. The dots represent the number of hotspots for birds, with denser dots representing a higher number of observed hotspots for birds.

A total of 1131 nocturnal bird hotspots were recorded in this survey, indicating the nocturnal roosting site preferences of campus bird species (Figure 3). Of these, 914 hotspots (81% of the total) were concentrated in areas B1, B4, and B5. Fewer birds roosted in street trees or other isolated trees within the campus. Seasonal variations significantly influenced the choice of nocturnal roosting sites. In spring, summer, and autumn, area B5 was the primary roosting site, while in winter, area B4 was preferred.

Analysis of the characteristics of bird‐preferred nocturnal roosts (Table 2) revealed that these sites were typically pure forests with a “tree‐herb” structure (B1, B3, B4), dominated by tall trees, a high degree of depression, and minimal hard‐surface cover in the understorey.

**TABLE 2:** Sample plot information.

Plot ID	Area (m^2^)	Tree species	Tree canopy cover (%)	Shrub cover (%)	Herbaceous cover (%)	Hard surface cover (%)	Canopy density (%)
B1	1400	*Ficus concinna* + *Cinnamomum camphora* + *Cyclobalanopsis glauca*	90	0	50	20%	95
B2	1400	*Ficus virens* + *Cinnamomum camphora* + *Ficus concinna* + *Cyclobalanopsis glauca* + *Magnolia* + *Alcea rosea* + *Lagerstroemia indica* + *Salix*	60	0	0	100	65
B3	1000	*Ficus virens* + *Cyclobalanopsis glauca* + *Broussonetia papyrifera*	86	40	0	20	90
B4	1000	*Ficus concinna* + *Livistona chinensis* + *Erythrina* + *Cyclobalanopsis glauca* + *Melia azedarach*	69	40	55	50	75
B5	1100	*Ficus virens* + *Cinnamomum camphora* + *Davidia involucrata*	40	30	0	40	47
B6	1100	*Cyclobalanopsis glauca* + *Ficus concinna* + *Ficus virens* + *Ginkgo biloba* + *Magnolia denudata* + *Davidia involucrata*	80	75	0	68	85
B7	1100	*Cedrus deodara* + *Ligustrum lucidum* + *Magnolia grandiflora* + *Ficus virens* + *Cyclobalanopsis glauca* + *Broussonetia papyrifera*	40	8	37	55	40
B8	1100	*Magnolia* + *Ficus virens* + *Cedrus deodara* + *Caryota urens* + *Cinnamomum camphora* + *Areca catechu* + *Cyclobalanopsis glauca* + *Prunus serrulata*	90	10	50	40	94

### 3.2 Plant Species Preferences for Birds' Nighttime Roosts

A total of nine nocturnal roosting plant species, spanning eight families and eight genera, were recorded in Area B of Chongqing University. These included *Ficus concinn*a, *Ficus virens*, *Magnolia grandiflora* , *Osmanthus fragrans* , *Cinnamomum camphora* , *Bischofia polycarpa*, *Bambusoideae*, *Ilex chinensis*, and *Melia azedarach* . *Ficus concinn*a and *Ficus virens* were the primary choices for nocturnal roosting, attracting a total of 1050 birds hotspots, which represented 93% of all birds recorded (Table 3). Notably, birds roosted at higher elevations on these plants during spring and summer compared to autumn and winter.

**TABLE 3:** Seasonal distribution of nocturnal roosting hotspots by site (B1, B3, B4) and tree species.

Season	Site					Tree species										
	B1	B3	B4	Total	Mean ± SD	*Osmanthus fragrans*	*Magnolia grandiflora*	*Ficus concinna*	*Cinnamomum camphora*	*Ficus virens*	*Bischofia polycarpa*	*Ilex chinensis*	*Melia azedarach*	*Bambusoideae*	Total	Mean ± SD
Spring	34	34	84	152	50.67 ± 28.87	0	6	51	3	143	3	0	0	1	207	23 ± 47.89
Summer	128	55	326	509	169.67 ± 140.22	3	10	250	6	286	3	0	0	0	558	62 ± 117.18
Autumn	21	33	55	109	36.33 ± 17.24	13	0	50	2	103	8	1	0	3	180	20 ± 34.92
Winter	37	73	34	144	48 ± 21.7	9	7	69	1	97	0	0	1	2	186	20.67 ± 36.16

To assess the influence of plant species and season on the distribution of nocturnal bird roosting hotspots, we fitted a generalized linear model (GLM). The model showed a significant overall fit, indicating that the predictors jointly affected roosting site selection (Table 4). Plant species had a significant effect on hotspot selection (Likelihood Ratio *χ* ^2^ = 30.167, df = 8, *p* < 0.001), consistent with distinct preferences among tree species. These results imply that bird preferences for specific tree species were consistent across seasons.

**TABLE 4:** Generalized linear model effects test on the selection of nocturnal bird roosting hotspots.

Source	Likelihood ratio *χ* ^2^	df	*p*
Intercept	11.840	1	0.001
Species	30.167	8	< 0.001
Season	5.019	3	0.170
Species × Season	17.018	14	0.255

*Note:* Dependent variable: Hotspots; Model fit indices: AIC = 1106.144, BIC = 1192.669.

**FIGURE 4:**
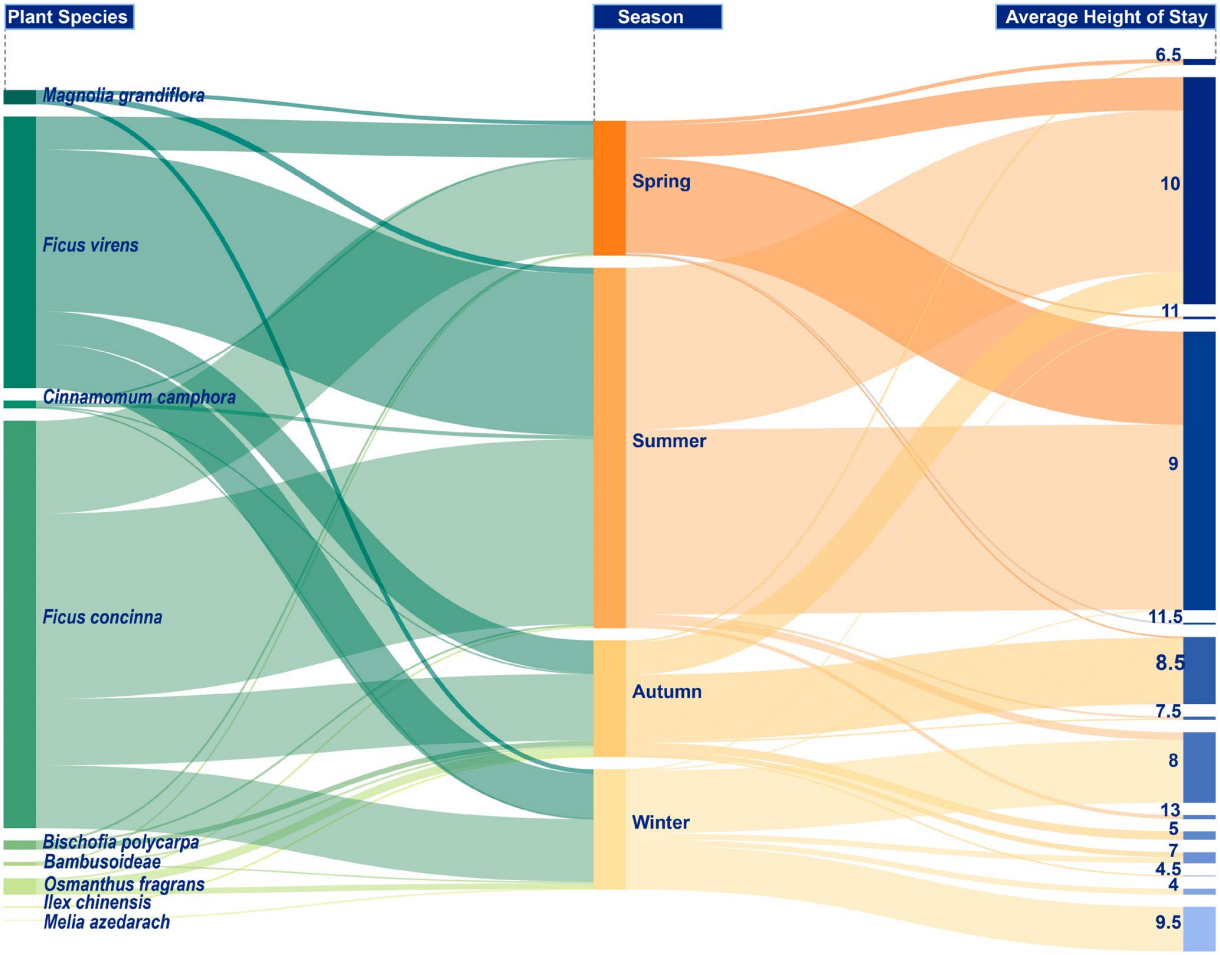
Associations between birds' nighttime roosts and roosting plant species. Sankey diagram linking plant species used by birds as nighttime roosts (left) to roosting height (m) (right), the width of the line indicates the number of bird hotspots, and the more hotspots that are recorded, the wider the line is.

Interestingly, birds were observed roosting at higher canopy positions during spring and summer compared with autumn and winter (Figure 4). This seasonal shift may reflect changes in microclimatic conditions or predation risk, although such effects were not captured by the species–season interaction in the current model.

### 3.3 Seasonal Differences in the Nocturnal Behavior of Birds

#### 3.3.1 Seasonal Differences in Site Selection for Birds at Nighttime Roosts

**FIGURE 5:**
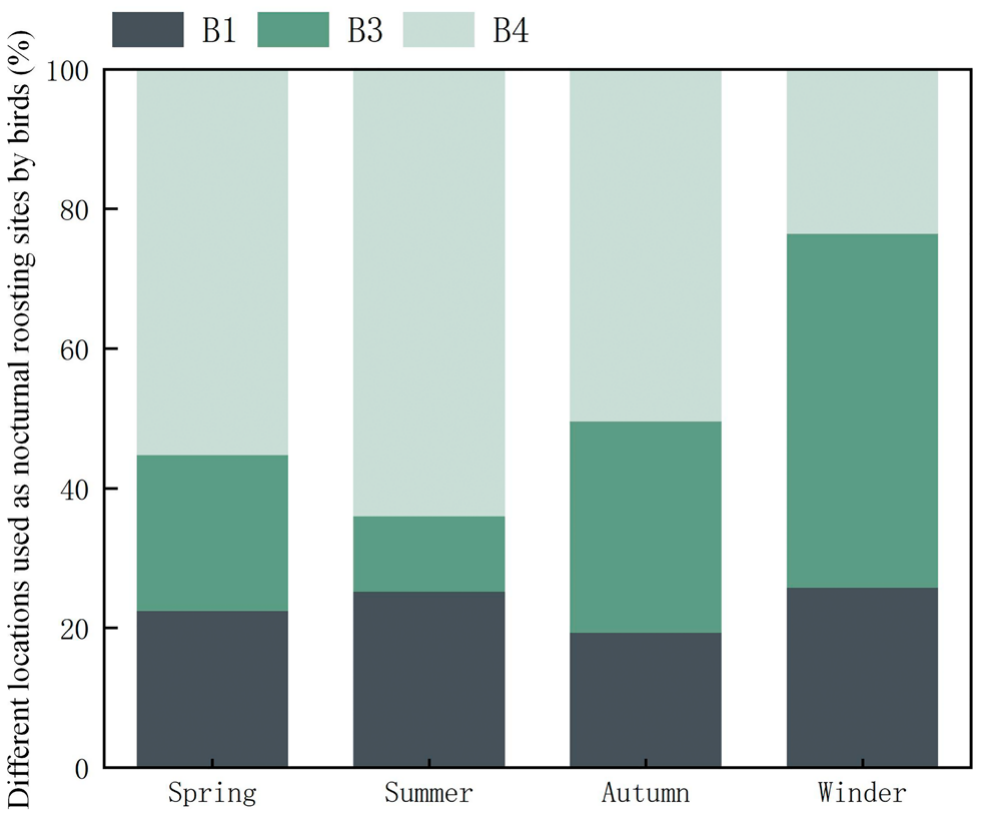
Proportion of birds' nighttime roosts across habitat types by season.

Bird selection for the main nocturnal roosts (B1, B3, and B4) varied seasonally (Figure 5). In spring and summer, B4 was highly preferred, reaching over 80% of selections in summer. In autumn, B4 usage declined while B3 increased to around 40%. By winter, B3 became the main roosting site, attracting 90% of birds, while B1 and B4 showed a marked decrease in selection.

#### 3.3.2 Seasonal Differences in the Height of Nocturnal Roosting Sites for Birds

**FIGURE 6:**
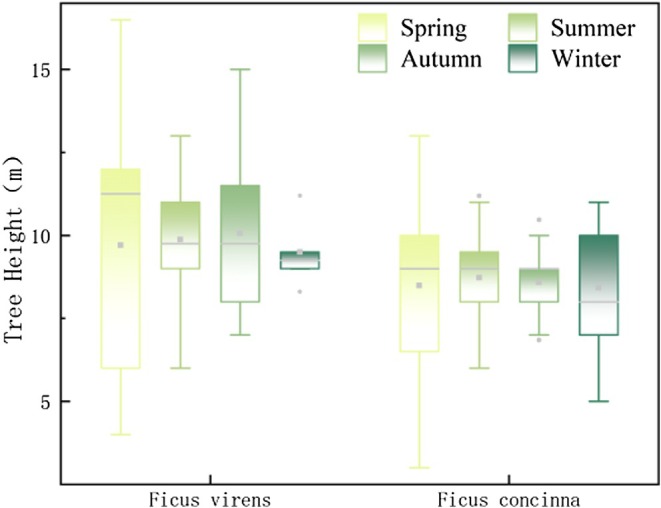
Bird nocturnal vegetation height box line plot. The vertical axis indicates tree height (M), while the horizontal axis distinguishes the two tree species. The box‐and‐whisker plots illustrate the range and median values of tree heights for each season.

Analysis of the two primary roosting species, *Ficus concinna* and *Ficus virens*, revealed significant seasonal variation in roosting height (Figure 6). One‐way ANOVA indicated a significant main effect of season (F = 2.512, *p* = 0.060). Due to a violation of the homogeneity of variances assumption (Levene's test, *p* < 0.05), Tamhane's T2 post hoc test was employed for pairwise comparisons. This analysis showed that the roosting height in winter was significantly lower than in summer (Mean Difference = 1.166, *p* = 0.032). Although not all pairwise comparisons with winter reached statistical significance, a general trend of lower roosting heights in winter was observed (Table S1).

**FIGURE 7:**
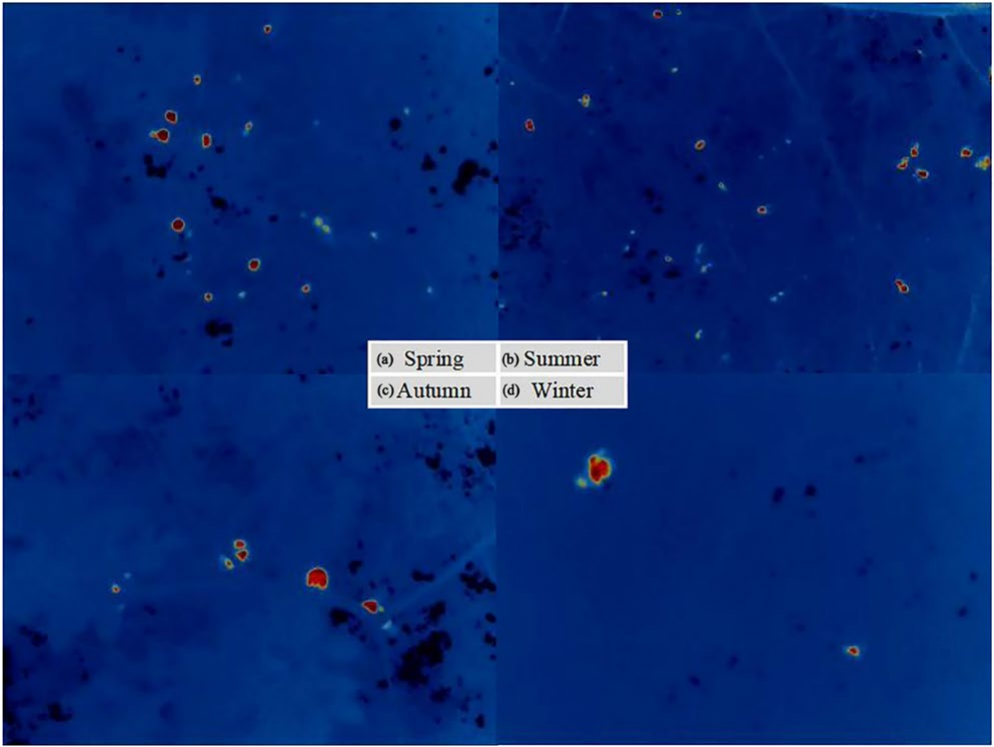
Seasonal patterns in nocturnal roosting hotspots. Panels show canopy‐level thermal detections of hotspots (red dots) for (a) spring, (b) summer, (c) autumn, and (d) winter. The observations indicate more dispersed hotspots in spring and summer and more clustered hotspots in autumn and winter.

Specifically, the distribution of roosting heights was widest in spring, particularly on Ficus virens, indicating more diverse height selectivity during this season. In contrast, roosting heights were more clustered in summer and autumn, reflecting more consistent height preferences. Thermal imaging observations corroborated these findings (Figure 7), showing that birds were more spatially dispersed in spring and summer, but formed denser clusters in autumn and winter.

## 4 Discussion

### 4.1 Habitat Preferences for Birds at Nighttime Roosts

Campus green spaces not only provide pleasant living environments for humans but also serve as essential habitats for urban birds. This study reveals distinct seasonal preferences in nocturnal roosting site selection by campus birds. Birds select their roosting sites carefully based on ecological needs and physiological traits, typically demonstrating strong site fidelity once a roost is chosen (Wang and Chu 2021). Across all seasons, approximately 81% of nighttime roost detections were concentrated in three consistently used plots (B1, B3, and B4). Specifically, plot B4, located in the retired faculty residential area with extensive vegetation cover, provided ample shelter and water resources, making it favorable for bird roosting. These results align with Yuan Baodong's findings (Yuan et al. 2018), which reported long‐term site fidelity in Hume's pheasant ( *Syrmaticus humiae* ). Notably, although B6 and B8 have high canopy cover (Table 2), both sites are dominated by *non‐Ficus* species, whereas hotspots in our study were concentrated in Ficus‐dominated plots. Comparative analyses (Table 2) suggest that this stable selection is associated with differences in tree height, plant assemblage, and the intensity of human disturbance, indicating that Ficus canopies with dense, horizontally tiered branches may provide more effective nocturnal shelter.

**FIGURE 8:**
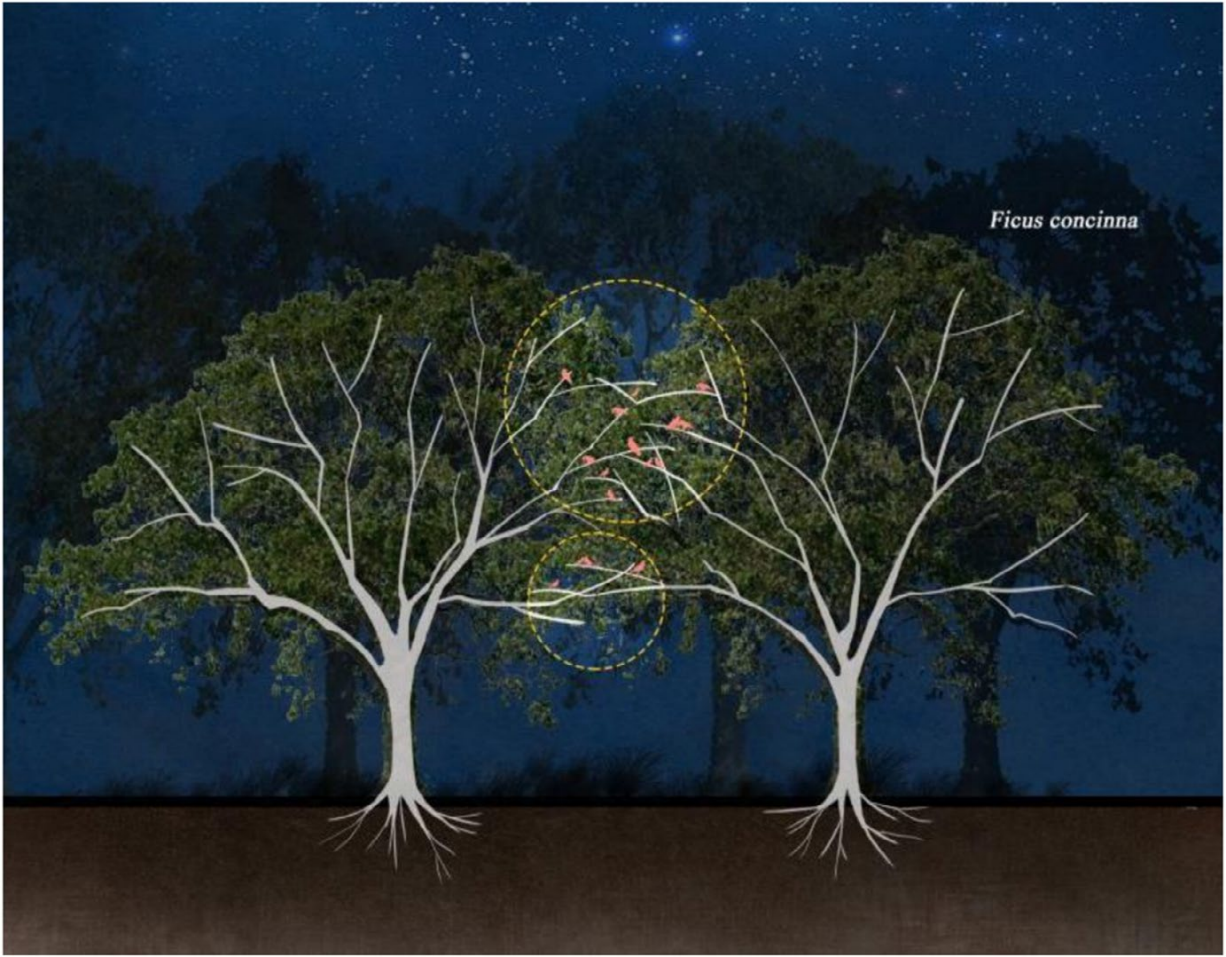
Schematic diagram of nocturnal roosting for birds. Dotted circles mark observed roosting birds within the canopy; most birds perch on outer branch tips in the upper middle crown, a position that likely reduces predation risk. Diagram is illustrative and not to scale.

Regarding habitat structure, analyses revealed that preferred nocturnal roosting plots (B1, B3, and B4) had higher canopy coverage formed by trees like *Ficus concinna* and *Ficus virens*, creating relatively safe roosting environments. Dense tree crowns offered birds protection and vigilance opportunities by minimizing predation risks and human disturbances. Notably, most birds preferred roosting at the junction of two trees (Figure 8), balancing visibility and ventilation while reducing susceptibility to disturbance.

Similar conclusions have been reported by Clergeau and Quenot (2007), who noted birds favor densely foliated trees accommodating multiple individuals, thereby enhancing predator defense capabilities. Pan et al. (2019) also found concealment of roosting branches critical for the safety of *Copsychus saularis* . Additionally, species like silver pheasants (*Lophura nycthemera*, Shao and Hu 2005) and barn swallows ( *Hirundo rustica* , Verma 2010) exhibit preferences for dense foliage and concealment. Species‐specific differences also occur, with some birds, such as blue‐eared pheasants ( *Crossoptilon auritum* ), favoring dense shrub habitats for increased ground‐level safety (Zhang et al. 2023). Consequently, varied tree heights or ground vegetation configurations in campus landscapes may lead to differentiated roosting patterns across bird communities, which accords with our observation that hotspots were concentrated in Ficus‐dominated plots with dense, horizontally tiered crowns.

In summary, this study's statistical analysis and quantified habitat assessments preliminarily confirm that tree‐herbaceous forest structures, high canopy coverage, minimal hardscape cover, and moderate concealment significantly influence nocturnal bird roosting preferences. Furthermore, the intensity of human disturbance also influences roost site distributions; quieter environments, like around plot B4, reduce birds' vigilance and enhance perceived safety. These findings provide empirical support for subsequent exploration of ecological functions and management optimization strategies in campus green spaces. By retaining key trees, maintaining canopy coverage, and controlling nocturnal human activities, campuses can better meet avian requirements for secure roosting habitats.

### 4.2 Seasonal Dynamics of Nocturnal Roosting Behavior

The study indicated that while birds often demonstrate fidelity to specific roost sites, bird abundance varied significantly across seasons. For example, bird numbers peaked in summer (breeding season), while roosting distributions differed during spring, autumn, and winter.

#### 4.2.1 Seasonal Distribution Patterns of Nocturnal Roosting Locations

High summer temperatures necessitate thermoregulation for birds, causing dispersion into more open, ventilated vegetation areas to reduce heat stress and energy costs related to thermoregulation (Zhao et al. 2023). According to heat‐balance theory, birds select roost sites dynamically to optimize local microclimates and minimize metabolic expenditures (Walsberg 1986). Empirical evidence supports this theory, such as spotted owls ( *Strix occidentalis* ) preferring sparse canopies with good airflow during hot months to mitigate heat stress (Barrows 1981), and Eurasian magpies (*Pica pica*, Crosbie et al. 2006) displaying individual roosting patterns correlated positively with temperature, reducing thermal interactions (Crosbie et al. 2006). Compared with typical urban green spaces, university campuses feature higher vegetation coverage and diversity, offering abundant microhabitats and behavioral flexibility crucial for mitigating thermal stress and optimizing resource acquisition (Sanllorente et al. 2023).

As temperatures decrease, birds face physiological pressures for heat retention, often forming communal roosts or selecting highly concealed environments. Studies on brown‐headed cowbirds (*Molothrus ater*, Francis 1976) indicate communal roosting reduces heat loss and enhances predator vigilance. Although oriental magpie robins (Pan et al. 2019) typically roost alone, occasional communal roosting occurs under low temperatures. Hence, clustered roost distributions during autumn and winter on campus green spaces represent adaptive responses to cold conditions.

#### 4.2.2 Seasonal Adjustments in Tree Species Preferences and Roosting Height

Trees like *Ficus virens* and *Ficus concinna* attract birds to varying degrees seasonally, influenced by leaf shedding cycles and vegetation density. For example, leaf shedding in spring reduces canopy density at plot B3, temporarily diminishing its attractiveness until foliage regeneration in winter restores its status as a hotspot.

Regarding roosting height, birds generally prefer relatively elevated positions but avoid the highest canopy points (Sedgeley 2001), balancing microclimate conditions and predator avoidance (Harris and McMurry 2024). Observations indicate average roosting heights were higher in spring, summer, and autumn compared to winter (Figure 5), potentially related to seasonal temperature variations and dynamic canopy structures. Kelty and Lustick (1977) observed similar patterns, with birds exploiting upper canopies for cooling in warmer seasons and lower branches during cold months to reduce heat loss.

In conclusion, seasonal dynamics in nocturnal roosting behavior are shaped by multiple ecological factors, including thermal balance, reproductive scheduling, and vegetation structure. Because our nocturnal protocol focused on thermal detections in trees and shrubs, ground‐dwelling or low‐roosting species (e.g., *pheasants*) may have been underdetected. Future work should incorporate ground‐level and understory assessments to better capture these taxa. Campus green‐space management strategies that account for these seasonal patterns by enhancing tree and shrub‐layer configuration could substantially strengthen the ecological services of campus environments, particularly roost provision and avian biodiversity conservation.

### 4.3 Human Disturbance and Birds at Nighttime Roosts Behavior

Despite environmental disturbances like artificial lighting at plot B3 and periodic academic bell sounds near plot B4, no significant impacts on bird roosting were detected. Previous studies indicate urban green spaces, often characterized by simple vegetation structures and limited diversity, force birds into restricted habitats where they adapt gradually to disturbances such as noise and lighting (Liu et al. 2023). Hence, noise and illumination are typically secondary disturbances for roosting birds. For instance, oriental magpie robins tolerate urban noise levels exceeding 40 dB (Pan et al. 2019), while rainbow lorikeets (*Trichoglossus moluccanus*, Daoud‐Opit and Jones 2016) prefer brightly illuminated areas, exemplifying urban bird resilience to disturbances.

Nevertheless, bird tolerance to disturbances has limits (Gorenzel and Salmon 1995). Despite suitable habitats, plots B7 and B8 near sports facilities showed significantly reduced roosting bird numbers due to persistent high human activity and late‐night illumination. This underscores that continuous or intense human disturbances negatively influence bird roosting behavior.

Future research should quantitatively assess disturbance thresholds through noise monitoring and human traffic analyses. Moreover, potential understudied disturbances like vehicle traffic and wildlife interactions require further exploration to comprehensively evaluate impacts on avian roosting behavior.

### 4.4 A Green Space Design Strategy for University Campuses Based on the Conservation of Nocturnal Roosting Sites for Birds

University campuses serve not only as vital spaces for study, research, and residence but also as essential ecological areas where humans and nature coexist harmoniously. Studies indicate that engaging in physical activities in green environments can significantly alleviate depression and stress (van den Bosch and Meyer‐Lindenberg 2019), particularly on campuses where academic and work pressures are prevalent. Thus, strategic planning and placement of green spaces across campus can help alleviate psychological stress. Additionally, campuses support substantial biodiversity, particularly in bird species richness (Ahir and Singh 2024). Campus green spaces provide essential bird habitats and play a key role in sustaining bird populations (Table S2, Zhang et al. 2023). However, some urban‐tolerant species frequently use campus roosts and may pose management challenges. Prolonged communal roosting can lead to guano accumulation that soils vehicles along roads and in parking areas, and vocal activity can disrupt study environments (Barcan and Johnston 2024). In response, this study proposes green‐space optimization strategies (Table 5) that balance human use with habitat preservation and foster a symbiotic campus ecosystem. Where intervention is necessary, actions should prioritize nonlethal, habitat‐based measures, such as periodic pruning scheduled outside the breeding season, selective crown lifting near entrances and roadways to discourage large aggregations, and routine cleaning, rather than removals that could cause avoidable ecological harm.

**TABLE 5:** Green space design strategies for university campuses.

Area	Strategies	Optimization module diagram	Goals
Academic and student living	Retain existing trees; add diverse shrub/herb layers—Prune trees near teaching buildings and study areas—Zone green spaces and walkways	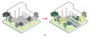	Guide pedestrian flow, reduce disturbance to bird habitats—Minimize bird song interference in study areas
Staff residential	Keep large‐canopy trees; mix evergreen and deciduous—Limit branch pruning—Preserve natural vegetation	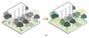	Provide shelter and nesting sites—Maintain seasonal resources and roosting stability—Enhance biodiversity
Sports	Increase open areas, separate activity zones from habitats—Use low‐attractiveness plants for birds	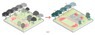	Reduce bird disturbance from human activity—Ensure cleanliness of sports facilities

For routine maintenance, it is recommended to retain perching branches of various sizes and heights to support birds' nocturnal concealment and roosting needs. Meanwhile, low branches near campus roads, parking areas, and entrances should be moderately pruned to limit bird congregation and reduce guano accumulation. Additionally, faculty and students from related disciplines are encouraged to participate in ecological monitoring and maintenance programs to assess vegetation growth and bird activity on campus, ensuring green spaces meet bird roosting needs while maintaining a clean campus environment. This process also provides valuable educational opportunities for students and promotes the harmonious coexistence of humans and nature.

## 5 Conclusions

Campus green spaces, as crucial ecological areas in urban settings, not only create a pleasant environment for learning and living but also serve as essential bird habitats, offering key areas for foraging, breeding, and predator avoidance. Research indicates that birds' choice of nocturnal roosts is stable and largely influenced by habitat characteristics; while roosting behaviors vary significantly with the seasons, reflecting birds' adaptability to environmental changes. Campus green spaces not only provide suitable habitats for birds but also significantly enhance bird diversity and support campus and urban biodiversity. Therefore, this study can help optimize the ecological functions of campus green spaces and provide a scientific basis for urban biodiversity conservation, supporting the healthy and sustainable development of urban ecosystems.

## Author Contributions

**Meng He:** conceptualization (equal), data curation (equal), investigation (equal), methodology (equal), writing – original draft (equal). **Bo Li:** funding acquisition (equal), investigation (equal), methodology (equal), writing – review and editing (equal). **Wei Du:** investigation (equal), visualization (equal). **Chunlan Du:** funding acquisition (equal), supervision (equal).

## Funding

This work was supported by the National Natural Science Foundation of China (Grant No. 52238003 and 52078074).

## Conflicts of Interest

The authors declare no conflicts of interest.

## Supporting information


**Table S1:** Pairwise seasonal comparisons of roosting height (m) for *Ficus concinna* and *F. virens* (Tamhane's T2, unequal variances).
**Table S2:** Daytime bird checklist for the campus.

## Data Availability

All the required data are uploaded as Supporting Information.
