# Supplementary material for: The Role of Campus as an Urban Multiuse Protected Area in Bird Nocturnal Roosting Habitat Function
Source: Ecol Evol. 2026 Jan 25;16(1):e72554. doi: 10.1002/ece3.72554 (PMC12832193; doi:10.1002/ece3.72554)
Supplement: Supplementary file 1 — Table S1: Pairwise seasonal comparisons of roosting height (m) for Ficus concinna and F. virens (Tamhane's T2, unequal variances). Table S2: Daytime bird checklist for the campus. [file ECE3-16-e72554-s001.docx]

## ****Supplementary Table S1.**** Pairwise seasonal comparisons of ****roosting height**** (m) for Ficus concinna and F. virens (Tamhane’s T2, unequal variances)

| **Comparison (I–J)** | **Mean difference (m)** | **SE** | **p** | **95% CI** |
| --- | --- | --- | --- | --- |
| Spring (1) – Summer (2) | −0.285 | 0.465 | 0.991 | [−1.530, 0.960] |
| Spring (1) – Autumn (3) | 0.743 | 0.556 | 0.708 | [−0.757, 2.243] |
| Spring (1) – Winter (4) | 0.881 | 0.475 | 0.338 | [−0.391, 2.152] |
| Summer (2) – Autumn (3) | 1.028 | 0.502 | 0.241 | [−0.337, 2.392] |
| **Summer (2) – Winter (4)** | **1.166** | **0.411** | **0.032** | **[0.064, 2.267]** |
| Autumn (3) – Winter (4) | 0.138 | 0.511 | 1.000 | [−1.250, 1.526] |

**Notes:** One-way ANOVA for season on height: F(3, 202) = 2.512, p = 0.060. Levene’s test indicated unequal variances (p < 0.05); therefore Tamhane’s T2 was used. Positive differences indicate the first season had a **higher** mean height than the second. Season codes: 1 = Spring, 2 = Summer, 3 = Autumn, 4 = Winter.

| **Supplementary Table S2. Daytime bird checklist for the campus.** | | |
| --- | --- | --- |
| Scientific name | Residence type | Characterisation |
| *Pycnonotus sinensis* | Resident | Urban-common |
| *Lonchura striata* | Resident | Urban-common |
| *Alcedo atthis* | Resident | Non-urban common |
| *Aegithalos concinnus* | Resident | Non-urban common species |
| *Urocissa erythroryncha* | Resident | Urban-common/Potentially nuisance |
| *Spodiopsar cineraceus* | Winter visitor | Urban-common/Potentially nuisance |
| *Copsychus saularis* | Resident | Potentially nuisance |
| *Passer montanus* | Resident | Urban-common |
| *Spodiopsar sericeus* | Resident | Urban-common/Potentially nuisance |
| *Turdus mandarinus* | Resident | Urban-common/Potentially nuisance |
| *Spilopelia chinensis* | Resident | Urban-common/Potentially nuisance |
| *Zosterops japonicus* | Resident | Urban-common |
| *Pterorhinus sannio* | Resident | Urban-common/Potentially nuisance |
| *Eophona migratoria* | Winter visitor | Non-urban common species |
| *Aegithalos concinnus* | Resident | Non-urban common species |
| *Pycnonotus xanthorrhous* | Resident | Urban-common |
| *Copsychus saularis* | Resident | Urban-common |
| *Hierococcyx sparverioides* | Summer visitor | Non-urban common species |
